# The benefits and risks of nostalgia: analysis of a fictional case with special reference to ethical and existential issues

**DOI:** 10.1186/s13010-023-00132-w

**Published:** 2023-04-19

**Authors:** Emmanuel Bäckryd

**Affiliations:** Pain and Rehabilitation Centre, and Department of Health, Medicine and Caring Sciences, Linköping University, Linköping, SE-581 85 Linköping Sweden

**Keywords:** Insomnia, Ethics, Existential, Nostalgia, Suffering, Virtues

## Abstract

**Background:**

In a previous paper in *Philos Ethics Humanit Med*, the 1937 Swedish novel *Sömnlös* (Swedish for sleepless) by Vilhelm Moberg was used as background for a thought experiment, in which last century’s progresses concerning the safety of sleeping pills were projected into the future. This gave rise to a theoretical discussion about broad medico-philosophical questions such as (among other things) the concept of pharmaceuticalisation.

**Methods:**

In this follow-up paper, the theme of insomnia in *Sömnlös* is complemented by a discussion of the concept of *nostalgia.* The core of the paper is a theoretical discussion about the benefits and risks of nostalgia, bringing together some aspects of recent psychological research about the construct of nostalgia with the main story line of the novel.

**Results and Conclusion:**

Nostalgia is portrayed as being, in some sense at least, ultimately beneficial for the protagonist of *Sömnlös*. This is congruent with recent psychological research. However, the story also shows that nostalgia may lead to problematic behaviours, at least when viewed from a virtue ethics perspective. Hence, nostalgia is both what leads the protagonist into ethically problematic behaviour and that which (paradoxically) ultimately saves him from his initial lack of courage, justice, temperance and practical wisdom. Moreover, the protagonist does not only “grow” ethically but also existentially. Hence, the novel opens up the possibility that insomnia and nostalgia might be viewed as bearers of important existential information (cf. sociologist of religion Peter L. Berger and his concept of “signals of transcendence”).

## Background

In his 1937 tripartite novel *Sömnlös* (Swedish for sleepless), Vilhelm Moberg portrays existential and relational distress in relation to insomnia. In a previous paper [3], I used the first part of the novel as a background for a thought experiment, in which last century’s progresses concerning the safety of sleeping pills were projected into the future. This gave rise to a theoretical discussion about broad medico-philosophical questions such as (among other things) the concept of pharmaceuticalisation. In the present paper, we will follow Knut Toring, the protagonist of *Sömnlös*, in parts 2 and 3 of the book. In so doing, we will explore the concept of nostalgia, with special reference to ethical and existential issues.

In Part 1, which is set in Stockholm, Toring has for some time suffered from insomnia. This is paralleled by an increasing sense of discontentment with life in general and work in particular, and with the materialism and pursuit of comfortableness that he associates with city life. He feels existentially disconnected, but his distress is also relational (his marriage is breaking down). Part 1 of *Sömnlös* ends with Knut Toring quitting work in Stockholm. He leaves his family and returns to Lidalycke, the small rural village in Southern Sweden where he grew up.

The relational and existential distress that Toring experiences, and which is correlated to insomnia, has a nostalgic dimension. Toring longs back to the village of his childhood. In the present paper, I will explore Toring’s return to Lidalycke from the point of view of nostalgia. I will first briefly summarize some central aspects of recent psychological research concerning the construct of nostalgia. Then, I will describe the main story line of Parts 2 and 3 of the novel in order to, third, bring together the two previous thought threads and explore what insights might be gained by weaving them together. Hence, the core of the paper is an analysis of Toring’s nostalgia, as depicted by Moberg, in the light of current nostalgia research. It will be argued that nostalgia is portrayed as being, in some sense at least, ultimately beneficial for Toring. However, the story also shows that nostalgia may lead to behaviours that are not necessarily unproblematic from an ethical point of view. I will conclude by asking whether nostalgia and insomnia can be viewed as bearers of valuable existential information, thereby also reconnecting with my previous paper [3].

### What is nostalgia?

When the term nostalgia was coined by Hofer in 1688, it designated a state of homesickness (*nostos*, return to the native land and *algos*, pain) [4], i.e., nostalgia had a geographical connotation. Semantic drift has broadened the meaning of nostalgia, and its commonplace dictionary definition now encompasses not only geography but also time – nostalgia as a bittersweet emotion about the past. It was initially a disease construct in the fields of medicine and psychiatry [4]. Although recent research has revived discussions about its potential negative effects [5], nostalgia is nowadays often considered to be a healthy psychological phenomenon [17]. For instance, it has been called “an important resource for psychological health and well-being” [14]. And according to Batcho [5], psychology research suggests that nostalgia has multiple beneficial effects such as strengthened social connectedness; continuity of self; enhanced self-esteem; adaptive coping strategies; meaning in life; and comfort in the face of one’s mortality (Batcho [5]; van Tilburg et al. [19]. We will come back to this impressive list of potential benefits later in the paper. Hence, nostalgia can be viewed both from a pathological and a salutogenic perspective, i.e., as a problem/risk or as a resource for health and well-being.

In the present paper, nostalgia will mainly be viewed from a psychological perspective. It is important to underline that this is far from the only way in which nostalgia can be studied. For instance, an issue about nostalgia in a *cultural studies* journal provided critical “entry points” as diverse as “history, film, popular music, African-American, Russian, and American Indian literature” [15]. Indeed, what Scanlan [15] says about “cultural critiques” should arguably be true for scholars and researchers from all kinds of academic fields:

[N]ostalgia was not, and is not, simple. It can cross several registers simultaneously. It can be felt culturally or individually, directly or indirectly. Indeed, cultural critics are beginning to understand that nostalgia is always complicated – complicated in what it looks like, how it works, upon whom it works, and even who works on it (Scanlan 2004) [15].

The psychological construct of nostalgia is not easily defined from a formal point of view, but a few conceptual distinctions are important to bear in mind. Importantly, *historical* nostalgia is different from *personal* nostalgia [4]; these are seen as relatively non-correlated phenomena. Simply put, personal nostalgia is about “the way *I* was”, whereas historical nostalgia is about “the way *it* was” (even before the person was born) [10]. A third concept, *anticipatory* nostalgia, has recently been introduced. In the words of Batcho [5]: “When nostalgia focuses on loss that has not yet occurred, the sadness of anticipated loss is premature and the experience becomes a paradoxical phenomenon of enjoying the present while missing it as if already relegated to the past. …. Anticipatory nostalgia depends upon mentally creating an imagined future that gives rise to missing what will be “someday past,” yet still present. By engaging abstract construal, anticipatory nostalgia might engender psychological distance from the present, decreasing direct involvement in the current concrete reality”.

## Methods

### The main story line of parts 2 and 3 of “Sömnlös”

Knut Toring has now come back to the village of Lidalycke [11]. Initially, he withholds information about the true nature of his visit; his relatives and the villagers think that he is simply on vacation. But gradually the truth is revealed, and Toring’s old parents are devastated. Toring has come back poor, he is out of job and is a would-be divorcee. Why? they ask him. He can’t really explain. It is not possible for him to put it into words.

Toring is surprised by how much the village has changed in recent years. Nostalgia for the agrarian society of his youth has led him back to the village of his ancestors, only to discover that this society no longer exists. The pursuit of comfortableness, which he fustigated in Stockholm, has found its way also to Lidalycke: electricity, new roads, cars, better housing, dietary changes. Even agriculture itself has evolved, the farmers now focusing on gains and productivity. Agriculture has become a business, relying not on the grace of God but on science and technology. The village of his youth no longer exists. Hence, the “spirit” in Lidalycke is now the same as in Stockholm, e.g. when it comes to money and greed. Indeed, the question is whether there has ever been any real difference; isn’t this simply human nature? [11] ^p. 300^. Moreover, and most ironically, it is precisely the remnants of this traditional and rural society that harshly condemn his intention to divorce his wife.

Toring still periodically suffers from insomnia. During harvest, hard work temporarily cures him, but his body gets used to hard work again and insomnia returns. His mother offers him traditional remedies (boiled milk, Hoffmann’s drops^1^). Realizing that the old village exists only within himself, Toring rents a small farm named Lyckemålen in the forest. And now finally he finds solace. Far from the village, in a kind of forestial hermitage, he finds traces of what he has been longing for. Most importantly, he begins reading again, notably the history of philosophy. Through rediscovering the reading passion of his youth, he builds up a kind of philosophy centred around the impossibility of finding meaning in life. There are no answers to the problems of life; they never go away. However, a key insight of his is that fighting these insolvable problems is what will keep him alive. The fight to stay aware of the impossibility of meaning, is itself meaningful. Existential distress is part of what it is to be human, and it will never disappear. He finds meaning in the words of Genesis 2:7: “Then the LORD God formed a man from the dust of the ground and breathed into his nostrils the breath of life, and the man became a living being.“ What matters is the living soul inside him.

But then Toring begins to feel lonely. He gets into a relationship with a young woman from the village (Jenny), but he cannot connect with her emotionally. He also begins to miss some aspects of modern life, notably electricity. And he misses his children. The relation with Jenny breaks down, and Toring realizes that he has somehow fooled himself. And then severe insomnia is back. Alone on his farm, he realizes that the most significant relationships in his life are those to his children – the bloodline is impossible to betray. It is a “contract in blood” [11] ^p. 422^. He also thinks about death, realizing that “love for life stems from the feeling of annihilation’s closeness” [11]^p. 415^. Reading a newspaper interview with (presumably) Hitler (the story is set in the 1930s), he ponders the enormous amount of suffering in the universe. His thoughts revolve around God’s role in this, and about the fact that the God of traditional Christianity *sleeps* (cf., the question of insomnia).

On a more positive note, he discovers that a small library has been started by a young woman in the village. There is hope! His sleeping is mixed: some nights are good, some are characterized by insomnia and thoughts about his children. He nonetheless begins to feel at ease again, as he is now being faithful to the spirit within him. He comes back to his earlier insight [11] ^p. 465^: “You never solve your problems, but perhaps you can get used to them. People never solve their problems, but they keep alive by fighting against them.” He acknowledges he was wrong in rejecting technology, which is indeed important, but only as a *means*. The important thing is to be free and not let work rob you of your dignity. And now Toring finds a kind of faith. Not in the God of his forefathers, not in the God of the Bible, but in a more immanent god, the god of growing and life, a god that does not need any church or preachers. And now finally, his inner emptiness is filled. The book ends with the finalization of his divorce (enforced by his wife and not by Toring) and the visit of his daughter for the summer holidays.

## Results

We will now look at Toring’s nostalgia from the point of view of contemporary nostalgia research. First, what kind of nostalgia does Toring experience?

### Personal, historical or anticipatory nostalgia?

Although there is an element of *personal* nostalgia in Toring, for instance in his longing to once again be an avid reader (which he was in his youth but paradoxically lost due to his work as an editor in Stockholm), Toring mainly experiences *historical* nostalgia. It is rural vs. urban, tradition vs. modernity, bloodline vs. individualism, soil vs. technology. Disappointed with city life in Stockholm, Toring longs for the old traditional way of life – only to discover it is on the verge of disappearing also in Lidalycke. And the little that is left of it will soon disappear – i.e., there is also a sense of *anticipatory* nostalgia [5].

Before going further in our analysis of the potential beneficial effects of nostalgia in Toring’s life, a short note on the names Lidalycke (the village) and Lyckemålen (the farm) is warranted. Given the whole context of the novel, it is perhaps not a coincidence that the fictional name of the village is Lidalycke, which literally means suffer-happiness (*lida*, to suffer, and *lycka*, happiness).^2^ There is a fair amount of suffering depicted in the novel, but the protagonist also finds a way to, if not happiness, at least some kind of contentment in life. The name Lyckemålen is also interesting, *målen* literally meaning “goals” in modern Swedish.^3^ So what kind of happiness does Toring find, and is it a consequence of nostalgia? And in what sense can happiness be at goal in life?

### The benefits of nostalgia?

Using the above-mentioned list provided by Batcho [5], I will now explore the potential benefits of nostalgia, as described by Moberg.

Strengthened social connectedness is the first beneficial effect listed by Batcho [5]. How can that be? The nostalgic individual populates the past with close others, and thereby it is thought that a feeling of social connectedness is nourished. For Toring, this is ultimately the case, as nostalgia initiated a process that eventually led to his reconnecting with his children. It is as if his geographical exile (fleeing Stockholm) was an outward sign of what was already the case in Stockholm – his inability to relate to his children. But, while away in Lidalycke, he finally realises that the force of bloodline relations extends not only backward in time but also forward, to his children. Hence, it seems that for Toring nostalgia, or rather the behaviour that was driven by his sense of nostalgia, has long-term beneficial consequences on social connectedness.

Next, continuity of self. According to this line of thought, nostalgia unifies our sense of who we are (our identity) over time, the connection to the past also helping us to see what we want to be in the future. This also seems to apply to Toring and his sense of identity. In the beginning of the novel, he does not seem to know who he is any longer. Nostalgia (or the behaviour driven by nostalgia) reconnects him with his past, thereby also giving a new orientation for who he will be in the future.

Third, enhanced self-esteem. Toring is highly critical of himself. He feels like a failure. At some point, he even says that he has fooled himself. But nostalgia has nonetheless created a process in which he finds his own self again (as mentioned above), and thereby he seems to gain an enhanced sense of self-esteem.

Adaptive coping strategies is next on the list. I would here like to mention the stress and coping model, which is important in the field of behavioural medicine [7]. When exposed to a stressor, the individual has a need to act in a way that restores homeostatic balance. Initially, Toring flees from Stockholm, i.e., he tries to avoid his problems by fleeing from them. In that sense, his sense of nostalgia is hardly constructive. But back in Lidalycke, a process of more adaptive coping starts, and eventually he learns to tackle problems head on instead of fleeing from them. Nostalgia has set forth a movement, that eventually leads to homeostatic balance. Toring finds that balance is not about solving your problems but learning to live with them.

Now to meaning in life, i.e., we now turn to explicit existential issues. I think I have made clear that Toring wrestles with such questions. Nostalgia drives him back home and somehow forces him to dig deep within himself in search for some kind of purpose. He then finds a paradoxical meaning in meaninglessness; there is purpose in the fight itself. Moreover, the pursuit of intellectual achievements is essential, and this is symbolized by books and the new library in the village.

Finally, Toring finds comfort in the face of mortality. He does so, it seems, in two ways. On the one hand, he finds solace in the thought that he will somehow continue to live through his children; cf. the first point about social connectedness. But on a more religious note, driven by nostalgia, he eventually finds some kind of faith. Not the one of his ancestors, but still a faith that is existentially helpful. There seems to be a high dose of Cartesian dualism in the way Moberg depicts the human condition, i.e., Toring’s anthropology is clearly dualistic (body vs. soul/spirit). Given Moberg’s own worldview (he was an atheist), this may seem rather unexpected.

### Can acting on nostalgic impulses be ethically problematic?

In the previous section, I repeatedly stated that Toring’s nostalgia, or rather the behaviour driven by nostalgia, eventually lead to effects that are mentioned in the literature as beneficial effects of nostalgia. In that sense, the novel’s description of nostalgia is congruent with recent psychological research about this construct. But of course, Toring’s behaviour can be ethically challenged. Hence, I will now discuss the ethics of nostalgia; or, more precisely, not the ethics of having or not having this feeling, but the ethics of how one reacts to this emotion.

My concise moral analysis of Toring’s behaviour will be made from the point of view of *virtue ethics* [2]. More precisely, I will analyse his behaviour from the point of view of the cardinal virtues of the classical tradition, i.e., courage, justice, temperance and practical wisdom [13]. These four traits of character can be found both in Plato and Aristotle, and they have also been key concepts in traditional Christian moral theology (which added the three theological virtues of faith, hope and love to the list). I think that a virtue analysis of Toring’s behaviour can help us identifying some of the ethical problems there might be in acting too swiftly on nostalgic impulses.

The novel gives the impression that Toring flees from his life in Stockholm. The metaphor of flight when being confronted to something difficult brings to the fore the cardinal virtue of courage. It could be argued that in the face of his nostalgic feelings (which are fuelled by his existential and relational distress), his actions show that the virtue of courage is underdeveloped in him. If he had had more courage, he would have stayed in Stockholm to confront his problems there instead of fleeing from them. Conceptually, one should distinguish physical courage, psychological courage and moral courage [12]. Toring does not lack physical courage, but in a certain sense he could be viewed as initially lacking psychological and moral courage, or perhaps better, existential courage. The novel can perhaps be viewed as a kind of *bildungsroman* in which Toring grows up and becomes a person who has the courage to face his existential situation in a way that he could not do at the outset. But when the novel begins, Toring is 37, and the question then surfaces: Isn’t there a worrying lack of character in this man, in that he just follows his nostalgic impulses and simply leaves his family? Writing from a nursing science perspective, Numminen et al. identified seven core attributes of moral courage [12]. Applying them to Toring’s flight from Stockholm is illuminating: true presence, moral integrity, responsibility, honesty, advocacy, commitment and perseverance, and personal risk. This is in many ways the opposite of how Toring acts.

Furthermore, the virtue of justice might also have led him to stay in Stockholm. The question of justice as a virtue (and what it means) is philosophically complicated [9], but here I will simply follow Papouli [13] according to whom justice “aims at eudaimonia^4^ of others rather than ourselves”. Even though Toring’s relationship with his wife seems beyond repair, it takes him almost a year to realize how important his *children* are. What about their eudaimonia? Using virtue ethics language: Does Toring really act *justly* towards his family? Hence, in addition to courage, the virtue of justice can be seen as an important resource to mobilize when experiencing nostalgic impulses.

Third, a dose of temperance would perhaps not have been superfluous. Quitting your job and leaving your family the way Toring does can be viewed as the opposite of temperance, which is “the virtue of moderation in action” [13].

Finally, there is the virtue of practical wisdom, phronesis. This is “the master virtue that guides all others” [13]. Phronesis has to do with being able to choose the wisest course of action in a difficult situation; it is about discernment [2]. The fact that Toring cannot put his decision to leave Stockholm into words (he can’t explain it to his parents), seems to be an indication that he lacks phronesis. He does not seem to be able to “read” himself. More of the cardinal virtue of practical wisdom would perhaps have helped him to find better ways to deal with relational and existential distress than just fleeing from everything. On the other hand, the suffering entailed by his fleeing from Stockholm helps him in the end to attain a certain happiness; one is once again reminded of the concept of eudaimonia which, according to Aristotle, is the ultimate goal [13]. Eudaimonia is often translated as happiness, and the name “Lidalycke” (as explained above) is interesting to ponder in this context, *lida* meaning suffering and *lycka* meaning happiness. It seems as if, for Toring, the path to happiness had to go through suffering. Through suffering, he finally found practical wisdom, the lack of which had led him into suffering in the first place.

So it could be said that Toring grows not only in practical wisdom but also in temperance, justice and courage: he nuances his earlier views; offers a path of reconciliation to his wife (although she rejects it); begins to reconnect with his children; and dares to expose himself to the “big” existential questions of human life.

## Discussion

### Nostalgia and insomnia as “signals of transcendence”?

Perhaps it could be said that for Toring, in his state of distress at the start of the novel, nostalgia is both what leads him into ethically problematic behaviour and that which (paradoxically) ultimately saves him from his initial lack of courage, justice, temperance and practical wisdom. And it seems to me that Moberg also tells us that nostalgia is not only important from an ethical point of view; it may in some cases at least be bearer of important existential information.

In a previous paper, I discussed the portrayal of existential and relational distress in Moberg’s *Sömnlös* in relation to insomnia [3]. Here, the focus has been on nostalgia, Toring experiencing both. In the novel, although both phenomena are intertwined, they can be disentangled (cf., the fact that I have written two papers, not one). What then is the relationship between them? Importantly, it does *not* seem to be a simple matter of causality, the one leading to the other. Their relationship is more mysterious than that. I would argue that Moberg somehow succeeds in creating the impression that nostalgia and insomnia, though different (the former has some kind of semantic content, whereas the latter is about difficulties in performing a basic biological function), stem from a common source. What this “source” is, is not entirely clear, but it seems to be some nameless existential discontent with life in general, a discontent from which both insomnia and nostalgia spring forth. In the following, I will therefore briefly discuss existential aspects of the insomnia-and-nostalgia complex as a whole.

The question I am asking now, is whether insomnia and nostalgia sometimes can be interpreted as symptoms that point beyond themselves to some of the “big” questions of human existence. Moberg seems to answer yes to this question, and therein lies perhaps an *existential* benefit of both nostalgia and insomnia. As existential signposts, they may be valuable; one is here reminded of sociologist of religion Peter L. Berger and his concept of “signals of transcendence” [6]. It is true that one should not settle down in their thin shadows, because nostalgia can be akin to depression (a major depression is a dangerous thing) and insomnia is not a particularly healthy phenomenon [3]. *All* sadness is however not bad or pathological – in many ways, being sad is part of life, as is the experience of nostalgia. Nostalgia is a feeling, and as such it is neither good nor bad, but Moberg seems to be teaching us that nostalgia can point beyond itself and thereby make us aware of the “big” questions.

One such existential question is that of suffering. On the one hand, the novel implies that a good character (virtues) can spare humans a lot of suffering. On the other hand, and paradoxically, Moberg shows us how virtues can grow out of suffering. Can it even be said that sometimes suffering is necessary in that respect? And that we should therefore not too quickly separate the ethical (virtues) from the existential (suffering)?

## Conclusion

Of course, suffering should never be romanticized. Perhaps some good can sometimes come out of it, but there are also countless examples of what is generally viewed as meaningless suffering, e.g., what philosopher Marilyn McCord Adams called *horrendous* evils [1]. Toring ponders over this in the novel, not least in relation to the sufferings of war. And in this context, a final note on the novel’s title seems warranted. *Sömnlös* means sleepless in Swedish, but besides Toring’s own insomnia, there are references to *God* as sleeping. It is as if Toring, when pondering the suffering of mankind, addressed to God the age-old question of Psalm 44: “Why do you sleep?” Hence, the novel can also be read as the story of a man who is awake while God himself seems to be sleeping. To be human is like living in the dark, being awake at night without the possibility of sleep. But, given the sheer amount of suffering in the world, shouldn’t *God* instead be the insomniac? From that point of view, the novel invites the reader to reflect upon the so-called problem of evil in the philosophy of religion [18], as well as on the problem of the “silence” and “hiddenness” of God [8]. Is God really silent (perhaps because he does not exist?) or is the silence of God rather “a silence between the words of God” [16], i.e., “the silence of a God who speaks” [20]? Disease and suffering, and their literary description, do indeed direct our attention to such “big” existential questions. But that is of course the subject of a completely different paper.

## Data Availability

Not applicable.

## Footnotes

1 A solution of alcohol and ether.

2 This is not at all the *etymological* sense of either *lida* or *lycke* in place names; my point here is symbolical, not etymological. Both *lid* and *lycke* are not uncommon in place names, so in Swedish Lidalycke makes perfect sense as a village name.

3 The suffix *-måla* is quite common in place names in that particular part of Sweden, but etymology is *not* the point here.

4 Eudaimonia is often translated as happiness, ‘human flourishing’, ‘wellbeing’, or ‘good life’ [13].
